# Modified high-entry C1 screw placement for bilateral persistent first intersegmental artery in atlantoaxial instability: a case report

**DOI:** 10.3389/fsurg.2026.1893144

**Published:** 2026-06-24

**Authors:** In-Suk Bae, Hyoung-Joon Chun

**Affiliations:** 1 Department of Neurosurgery, Nowon Eulji Medical Center, Eulji University, Seoul, Republic of Korea; 2 Department of Neurosurgery, Hanyang University Medical Center, Hanyang University, Seoul, Republic of Korea

**Keywords:** atlantoaxial instability, C1 screw, persistent first intersegmental artery, rheumatoid arhritis, vertebral artery

## Abstract

**Background:**

Atlantoaxial instability (AAI) is a recognized cervical manifestation of rheumatoid arthritis (RA) and may require posterior C1-C2 fixation. Conventional C1 lateral mass screw placement can be hazardous when anomalous vertebral artery (VA) anatomy is present, particularly in patients with persistent first intersegmental artery (PFIA).

**Case description:**

A 70-year-old woman with long-standing RA presented with a 6-month history of nuchal pain, rotational neck pain, and occipital neuralgia. Plain radiographs demonstrated AAI with an atlantodental interval of 7.5 mm, and magnetic resonance imaging revealed widening of the C1–C2 interspinous space without spinal cord compression. Computed tomography (CT) angiography revealed bilateral PFIA, with both VAs ascending directly into the foramen magnum without traversing the transverse foramen of C1. Because this anatomy directly intersected the conventional C1 lateral mass screw trajectory, a modified high-entry point was selected at the junction of the posterior arch and lateral mass. C1 lateral mass-C2 pedicle screw fixation was performed without intraoperative vascular or neural complication, and postoperative CT confirmed satisfactory implant placement. Early postoperative neck pain and occipital neuralgia improved.

**Conclusion:**

In patients with RA-associated AAI and bilateral PFIA, the standard C1 lateral mass trajectory may be unsafe. This case illustrates that a modified high-entry C1 screw placement can be a feasible alternative when guided by careful preoperative vascular imaging and individualized surgical planning.

## 1 Introduction

Rheumatoid arthritis (RA) frequently involves the cervical spine and may lead to atlantoaxial instability (AAI), which carries a risk of myelopathy and vertebrobasilar ischemia (1). Posterior C1–C2 screw fixation is a commonly used technique for stabilizing AAI but poses a risk of vertebral artery (VA) injury, particularly in the presence of VA anomalies at the craniovertebral junction (2).

Several anomalous VA courses at the craniovertebral junction have been described and are broadly categorized into intraosseous variants, such as high-riding VA at the C2 isthmus, and extraosseous variants, including fenestration and persistent first intersegmental artery (PFIA). PFIA is a very rare condition, with a prevalence of 0.01% to 1.8% (3–5). When present, a PFIA may significantly alter the safe corridor for C1 screw placement. In this anomaly, the VA enters the spinal canal between C1 and C2 and ascends toward the foramen magnum without following the usual course through the C1 transverse foramen, thereby potentially crossing the standard C1 lateral mass screw trajectory.

We report a rare case of combination of RA-associated AAI, bilateral PFIA, and successful short-segment fixation using a modified high-entry C1 screw without extensive VA mobilization. The technical message is not that one method should be used routinely, but that preoperative vascular imaging can identify when a standard trajectory is unsafe and when a tailored alternative is required in selected patients.

## 2 Case description

A 70-year-old woman with long-standing RA presented with a 6-month history of nuchal pain, rotational neck pain, and occipital neuralgia. Plain radiographs revealed an AAI with an atlantodental interval of 7.5 mm. Magnetic resonance imaging revealed widening of the C1–C2 interspinous space without spinal cord compression.

Because posterior C1-C2 instrumentation was being considered, preoperative computed tomography (CT) angiography was obtained to assess both bony and vascular anatomy at the craniovertebral junction. Three-dimensional reconstructions demonstrated an anomalous course of both VAs at C1–C2; each VA entered the spinal canal between C1 and C2 and ascended to the foramen magnum without traversing the transverse foramen of the atlas, consistent with bilateral PFIA (Figure 1). The anomalous vessels coursed immediately caudal to the C1 posterior arch and lateral mass, directly overlapping the conventional entry zone and trajectory used for standard C1 lateral mass screws.

**Figure 1:**
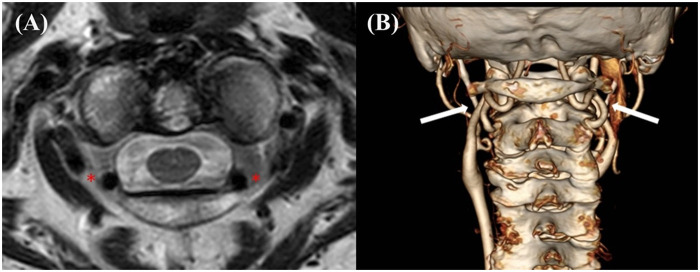
Preoperative imaging demonstrating bilateral persistent first intersegmental arteries. **(A)** Axial magnetic resonance image showing bilateral vertebral arteries adjacent to the thecal sac at the C1-C2 level. **(B)** Three-dimensional CT angiographic reconstruction demonstrating that both vertebral arteries enter the spinal canal between C1 and C2 and ascend directly toward the foramen magnum without traversing the C1 transverse foramen.

A modified high-entry C1 screw point was therefore planned at the junction of the posterior arch and lateral mass, with the screw trajectory directed along the posterior arch-lateral mass corridor to remain within a safer bony pathway while avoiding the anomalous arteries.

### 2.1 Operative technique

The patient underwent posterior C1-C2 fixation under continuous fluoroscopic guidance. After exposure of the posterior elements, particular attention was paid to the anomalous vascular anatomy identified on preoperative CT angiography. Full VA dissection and mobilization were intentionally avoided to reduce the risk of arterial injury in the setting of bilateral PFIA, because the anomalous vessels directly overlapped the conventional screw corridor.

At C1, the screw starting point was moved superiorly to the junction of the posterior arch and lateral mass rather than the conventional point inferior to the arch, where the anomalous arteries coursed immediately adjacent to the standard entry zone. Specifically, the modified entry high-entry C1 screw point was established at the superior margin of the C1 posterior arch where it merges with the lateral mass, approximately 2–3 mm lateral to the medial edge of the lateral mass (Figure 2). The screw trajectory was directed with a strict parallel alignment to the posterior arch in the sagittal plane (approximately 0°–5° cephalad) and angled approximately 10°–15° medially in the axial plane to target the thickest portion of the C1 lateral mass. This modification was chosen because the standard trajectory was obstructed by the caudally coursing anomalous arteries. The screw was directed along the posterior arch-lateral mass pathway to bypass the vascular conflict zone while preserving rigid C1 fixation and avoiding vertebral artery mobilization.

**Figure 2:**
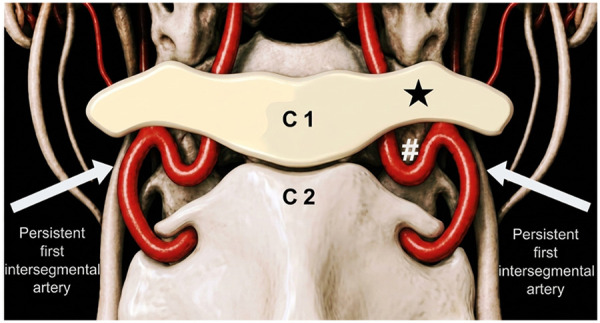
Illustration showing the modified high-entry C1 screw entry point, located superiorly at the junction of the posterior arch and lateral mass. The modified entry point is indicated by ★, and the conventional C1 lateral mass screw entry point, obstructed by the persistent first intersegmental artery, is indicated by #.

At C2, pedicle screws were inserted under fluoroscopic guidance, and rods were placed to complete C1-C2 stabilization. This strategy was selected over more extensive constructs, such as occipitocervical fusion (OCF), because a short-segment construct appeared achievable based on the patient's remaining osseous corridor and because preservation of motion segments beyond C1-C2 was desirable. This choice was made after weighing the available bony corridor against the risks of a longer fusion. No arterial bleeding, neural compromise, or other intraoperative complication was observed.

### 2.2 Postoperative course

Postoperative CT demonstrated satisfactory placement of the C1 and C2 screws without violation of the spinal canal, transverse foramina, or visible vascular structures (Figure 3). Early after surgery, the patient's nuchal pain and occipital neuralgia improved, and she was discharged without new neurological deficit. This report primarily documents technical feasibility and immediate postoperative safety rather than long-term construct performance.

**Figure 3:**
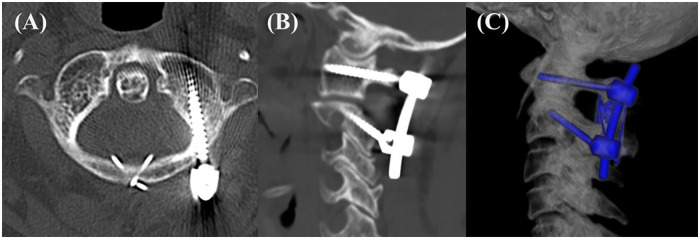
Postoperative CT images after C1-C2 fixation. **(A)** Axial CT view showing screw position relative to the spinal canal and transverse foramina. **(B)** Lateral CT view demonstrating the modified C1 screw trajectory. **(C)** Three-dimensional reconstruction showing the left C1 screw originating from the posterior arch-lateral mass junction.

## 3 Discussion

PFIA is a persistent embryological first intersegmental artery that fails to regress and instead continues to supply the V3 segment, resulting in an anomalous course of the VA at the C1–C2 level. It is a very rare condition representing between 0.01% and 1.8% (3–5). Most patients with this condition have a unilateral PFIA, but it occurs bilaterally in a small minority of cases (6). As the VA normally runs in the sulcus arteriosus over the superior aspect of the C1 posterior arch, most classical C1 lateral mass screw techniques aim for an entry point just inferior to the arch beneath the groove to maximize the purchase while avoiding the artery. However, in PFIA and related variants, such as when the VA bypasses the C1 transverse foramen or enters the spinal canal between C1 and C2, the artery may lie directly in line with conventional screw trajectories, making these techniques hazardous.

Previous studies have suggested that PFIA should be regarded as a relative or absolute contraindication to standard C1 lateral mass screw placement and may necessitate alternative strategies (7–9). In patients with AAI, particularly those associated with RA, vascular anomalies such as PFIA further narrow the safe bony corridor for instrumentation, especially in bilateral cases (10, 11). To mitigate these risks, alternative strategies include occipitocervical fusion (OCF), which provides robust stability for complex instability but sacrifices occipitoatlantal motion and increases overall morbidity. Another option is C1 posterior arch or laminar screw fixation, which offers biomechanical advantages, such as higher pullout strength and reduced venous bleeding or C2 nerve root irritation, when the posterior arch thickness exceeds 4 mm; however, this is often not feasible in RA because of erosive bone changes (12, 13). In contrast, the modified high-entry C1 posterior arch–lateral mass trajectory used in this case preserves motion segments distal to C1–C2, avoids the need for VA mobilization, and provides effective short-segment stability. These benefits are consistent with prior anatomical and biomechanical reports of supralaminar and posterior arch-based screw techniques in selected patients with anomalous VAs (14).

It is important to explicitly clarify the originality of our proposed high-entry approach relative to previously described supralaminar or standard posterior arch-based techniques. Traditional posterior arch or laminar screw fixation frequently utilizes the mid-portion or inferior edge of the arch as an entry corridor, which relies on a posterior arch thickness exceeding 4 mm. In contrast, our technique specifically targets the superior junction of the posterior arch and lateral mass. This high-entry point intentionally leverages the biomechanical stability of the dense lateral mass junction while bypassing the caudal-lying PFIA variant entirely. Unlike pure supralaminar techniques that run within the lamina itself, this modified trajectory acts as a structural bridge, making it uniquely reproducible in patients who exhibit erosive or thin posterior arches from RAs. Furthermore, both anatomical and clinical investigations indicate that these modified approaches can achieve secure and safe fixation, even in complex vascular variants, especially when guided by meticulous preoperative CT angiography and intraoperative verification (7, 15).

Potential advantages sometimes associated with posterior arch-based or modified high-entry techniques, such as reduced venous plexus bleeding, less C2 nerve root manipulation, or lower postoperative neuralgia, may be reasonable hypotheses, but they were not independently established by this single case. However, in patients with RA and degenerative cervical changes, a thin or eroded C1 posterior arch can make palpation, decortication, and screw placement technically demanding, so meticulous preoperative planning and intraoperative verification remain essential.

Beyond posterior fixation strategies, anterior decompression via an endoscopic endonasal route has emerged as an important option for irreducible basilar invagination and other ventral craniovertebral Junction (CVJ) pathologies. Previous studies have shown that endoscopic endonasal odontoidectomy can achieve effective anterior decompression with favorable clinical outcomes, but often requires staged or complementary posterior occipitocervical stabilization (16–18). In that context, careful preoperative assessment of VA variants is important because these anomalies may constrain not only conventional C1 screw trajectories but also posterior fusion options after anterior decompression, reinforcing the need for anatomy-driven planning at the craniovertebral junction.

The principal technical contribution of this case is the use of a modified high-entry starting point at the posterior arch–lateral mass junction to avoid the vascular conflict zone while still enabling short-segment C1-C2 fixation. Unlike OCF, this approach maintains occipitoatlantal motion and avoids the morbidity associated with more extensive fusion. Although prior studies have described posterior arch-based and laminar screw techniques in the setting of anomalous VAs, the value of the present technical report lies not in the anomaly alone, but in the combination of RA-associated AAI, bilateral PFIA, and successful fixation using a tailored C1 high-entry approach without extensive VA dissection.

## 4 Study limitations and future outlook

This report primarily documents technical feasibility and immediate postoperative safety rather than long-term construct performance. Because follow-up was limited, no definitive conclusions can be drawn regarding long-term fusion rates, construct durability, or radiological consolidation beyond the early postoperative period. The lack of multi-year clinical and radiological outcomes remains a limitation of this technical case report. While early postoperative imaging confirmed excellent screw purchase and complete decompression, future prospective or registry-based studies with extended follow-up are necessary to fully evaluate long-term fusion rates, potential implant subsidence, and late construct failure modes associated with this high-entry trajectory in the rheumatoid spine.

## 5 Conclusion

This case report demonstrates that bilateral PFIA may make conventional C1 lateral mass screw placement unsafe in RA-associated AAI. Careful preoperative CT angiography can identify this hazard and support individualized modification of the C1 entry point and trajectory. In selected patients, a modified high-entry C1 lateral mass screw provides a reproducible and biomechanically sound alternative for achieving robust C1-C2 fixation while successfully avoiding anomalous VAs.

## Data Availability

The original contributions presented in the study are included in the article/Supplementary Material, further inquiries can be directed to the corresponding author.
